# Calculating the Risk of Admission to Intensive Care Units in COVID-19 Patients Using Machine Learning

**DOI:** 10.3390/jcm14124205

**Published:** 2025-06-13

**Authors:** Mireia Ladios-Martin, María José Cabañero-Martínez, José Fernández-de-Maya, Francisco-Javier Ballesta-López, Ignacio Garcia-Garcia, Adrián Belso-Garzas, Francisco-Manuel Aznar-Zamora, Julio Cabrero-García

**Affiliations:** 1 Grupo Ribera, Edificio Sorolla Center, Avda Cortes Valencianas, 58, 46015 Valencia, Spain; fjballesta@riberasalud.es; 2 Nursing Department, University of Alicante, 03690 San Vicente del Raspeig, Spain; mariajose.cabanero@ua.es (M.J.C.-M.); julio.cabrero@ua.es (J.C.-G.); 3 Vinalopó University Hospital, 03293 Elche, Spain; jfernandez@vinaloposalud.com; 4 Verne Technology Group, 03144 Alicante, Spain; ignacio.garcia.ds@gmail.com; 5 Ribera Salud Technologies, 03203 Elche, Spain; abelso@futurshealth.com (A.B.-G.); faznar@futurshealth.com (F.-M.A.-Z.)

**Keywords:** COVID-19, artificial intelligence, risk management, critical care, patient safety

## Abstract

**Background:** The COVID-19 pandemic clearly posed a global challenge to healthcare systems, where the allocation of limited resources had important logistical and ethical implications. Detecting and prioritizing the population at risk of intensive care unit (ICU) admission is the first step to being able to care for the most vulnerable people and avoid unnecessary consumption of resources by mildly ill patients. **Objective:** To create a model, using machine learning techniques, capable of identifying the risk of admission to the ICU throughout the hospital stay of the COVID patient and to evaluate the performance of the model. **Methods:** A retrospective cohort design was used to develop and validate a classification model of adult COVID-19 patients with or without risk of ICU admission. Data from three hospitals in Spain were used to develop the model (*n* = 1272) and for subsequent external validation (*n* = 550). Sensitivity, specificity, positive and negative predictive value, accuracy, F1 score, Youden index and area under the curve of the model were evaluated. **Results:** The LightGBM model, incorporating 40 variables, was used. The area under the curve obtained by the model when the test dataset was used was 1.00 (0.99–1.0), specificity 0.99 (0.97–1.00) and sensitivity 0.92 (0.86–0.98). **Conclusions:** A model for predicting ICU admission of hospitalized COVID-19 patients was created with very good results. The identification and prioritization of COVID-19 patients at risk of ICU admission allows the right care to be provided to those who are most in need when the healthcare system is under pressure.

## 1. Introduction

In December 2019, a new pneumonia (COVID-19) caused by the SARS-CoV-2 virus broke out in Wuhan (China). The most widespread symptoms were fever, dry cough, muscle weakness, and chest pain [1]. As of December 2021, the number of COVID-19 infections worldwide amounted to 290 million people and 5,400,000 deaths. In Spain, 6,290,000 people were infected and 89,000 died of the disease [2]. According to data collected from the beginning of the pandemic, 20% to 30% of COVID patients required hospitalization, and 5% to 12% needed intensive care [3]. The situation clearly posed a global challenge to health systems for various reasons, such as: rapid spread of the disease; high concentration of cases; excessive consumption of resources; high percentage of severe cases superior to other respiratory syndromes [4]; and high mortality rate of the most severely affected [5].

In scenarios of such huge pressure on health systems, allocating limited resources such as intensive care unit (ICU) beds has important logistical and ethical implications [6]. In other words, it is crucial to detect and prioritize patients in need of intensive therapy to avoid the unnecessary consumption of medical resources by mild or asymptomatic patients [7,8].

No clear prognostic biomarkers have hitherto been defined allowing to predict which patients will require ICU care. Indeed, many laboratory markers are affected by the disease and their presentation varies in terms of symptom severity or patient deterioration speed [9].

Nevertheless, multiple efforts have been made since the beginning of the pandemic to create tools based on artificial intelligence which help to screen, diagnose, and predict COVID-19 patient prognosis. These tools use radiological images and clinical laboratory data [10] because respiratory status, immune and inflammatory response as well as coagulation, among others, are significantly altered during the disease [11]. Most studies which have focused on probable disease evolution are based on different aspects of clinical deterioration as dependent variables, whether grouped or separated, such as ICU admission, severe symptoms, shock, mechanical ventilation needs, or patient death [3,4,7,12,13,14,15,16,17,18,19,20,21,22,23,24,25]. Most of the studies, which include the variable ICU admission or analogous outcomes (such as the need for invasive mechanical ventilation), present predictive models which rest on data relating to the patient’s first contact (static variables) with the hospital [7,14,15,16,17,18], but few consider the patient’s evolution (dynamic variables) during hospital stay [3,19,20,21]. The exclusive use of static variables prevents the model from being able to reflect the patient’s condition over the days and makes suboptimal predictions of poor value.

Based on the above, the objective of the study was to develop a predictive model of the risk of intensive care unit admission in patients with COVID-19.

## 2. Materials and Methods

### 2.1. Setting

The study was conducted with patients admitted to 3 hospitals in Spain, all of which were of medium size (200–250 beds) and had emergency, medical hospitalization, and ICU departments. All variables were retrieved from the electronic medical record (EMR).

### 2.2. Design

A retrospective cohort design was used to develop and validate the model for classifying patients with or without ICU admission risk. Data from the three hospitals were used to develop the model and for its subsequent external validation. Datasets from different time periods were used for each purpose (training and test).

### 2.3. Data Preparation

The eligible population met the following criteria: patients aged over 16 years who were confirmed COVID-19 positive by a laboratory (C-Reactive Protein (CRP) test, CRP rapid test, or antigen test) at the time of admission or over the previous 7 days, who presented a respiratory-type main diagnosis (COVID-19, SARS-associated coronavirus infection, infection due to unspecified coronavirus, unspecified pneumonia), as well as a hospitalization duration equal to or greater than 24 h (including a stay in the emergency department). Patients whose medical record included therapeutic effort limitation were excluded (17% of the total number of COVID-19-diagnosed patients).

**Figure 1:**
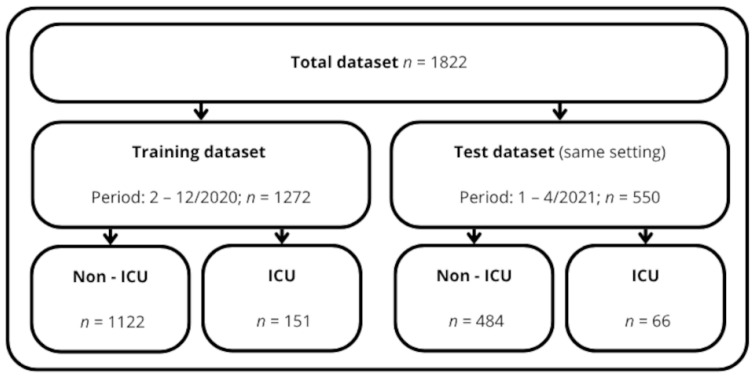
Design of the study.

The data collection period spanned from 15 February 2020 to 30 April 2021. The total sample included 1822 patients. The first dataset of the 3 hospitals was used to develop the model which corresponded to the sample collected between 15 February and 31 December 2020 (*n* = 1272). External validation rested on a dataset from the same hospitals but obtained over a different period, specifically between 1 January and 30 April 2021 (*n* = 550) (Figure 1).

### 2.4. Dependent Variable

The dependent variable was defined as the ICU admission of a patient coming from any service and having been previously hospitalized for more than 24 h. If the patient had been admitted more than once to that unit, only the first admission was included in the analysis. Those who were not admitted to the ICU and whose end-destination was discharge home, transfer to another center, or death were considered as non-ICU patients in this study.

### 2.5. Independent Variables

We conducted a review of the literature to identify ICU admission predictors in patients with COVID-19 and other pneumonias. A total of 96 variables were selected. Next, the possibility of obtaining these variables from the EMR was evaluated and the criteria for doing so were defined according to each variable, both for patients who were admitted to the ICU and for those who were not. The values of each variable were extracted based on different temporal strategies (on admission, during the stay, or at discharge). When the data of the variable were generated during the stay, the value was obtained at a single time for each subject at the time closest to ICU admission (or on the expected ICU admission day, median).

Of the total number of variables, the following were excluded before the imputation: 23 categorical variables, because they were already included as numerical variables; 30 variables, because they presented a high rate of null values (more than 10%); 7 due to high correlation with other variables already included; 5 variables using recursive feature elimination of variables (RFE) techniques; and 1 due to inaccessibility. Missing values were imputed with the median in continuous variables and with the mode in categorical variables. To handle outliers, the interquartile range (IQR) was applied. Additionally, categorical variables with response rates below 25% were recoded.

The extracted variables, to which we added new derived variables (created from relevant original variables) were processed to generate a database that we used to select the algorithm. The original variables which the model was finally based on are detailed in Appendix A.1.

### 2.6. Model Development

To select the predictive model algorithm, the training sample from February to December 2020 was used. The performance of four algorithms was evaluated: LightGBM, XGBoost, logistic regression and random forest. Recursive feature elimination cross validation (RFECV) was implemented to simplify the model by identifying the optimal number of variables without compromising its predictive capacity. To counteract the imbalance of the dependent variable, the Adaptive synthetic sampling approach for imbalanced learning (ADASYN) technique was applied. In addition, stratified k-fold cross validation was used, with k = 5, to prevent model overfitting and bias. The hyperparameters were optimized using the Bayesian search technique.

Once the final model was established, its performance was analyzed calculating sensitivity (the probability of the positive label being true), specificity (the probability of the negative label being true), positive and negative predictive value (the proportions of positive and negative results in tests that are true positive and true negative results, respectively), accuracy (probability of the true value of the class label), F1-score (harmonic mean of the model’s precision and sensitivity), Youden index (evaluates the algorithm’s ability to avoid failure), and area under the curve (AUC quantifies the ability of a model to distinguish between different classes), with their confidence intervals. An external validation using the test dataset was performed and Shapley additive explanations (SHAP) were used to improve model interpretability. The development was based on a machine learning framework in Python v3.7.9, using Scikit-Learn and LightGBM. “Azure Machine Learning” and ‘Microsoft Kubernetes’ were used to move from test environment to a production environment, available to end users.

## 3. Results

The model development was based on a sample of 1272 subjects, of which 12% were admitted to the ICU. The discharge of the remaining 88% who remained hospitalized was defined as follows: 71.73% were sent home; 21.63% were admitted to home hospitalization; 6.38% passed away; and 0.28% were transferred to another center. A total of 58% were men, and those aged over 60 accounted for 63% of the sample. Most of patients were Spanish (67%). They remained hospitalized for 208 h on average after admission and the most common respiratory therapy was nasal prongs. The median time elapsed to be admitted to the ICU was 72 h. The samples used to develop and validate the model presented significant differences across all variables except sex, place of birth, hours of hospitalization, hours of anticoagulant treatment, creatinine value, D-dimer value, ferritin value, PCO_2_ value, platelet value, and aspartate aminotransferase (AST) value (Table 1).

The performance of the four machine learning models studied is detailed in Table 2. LightGBM was the selected algorithm because it presented the best metrics (the higher the numerical value, the greater the predictive capacity).

The final model was composed of a total of 40 variables, 30 of which were original and 10 derived.

**Figure 2:**
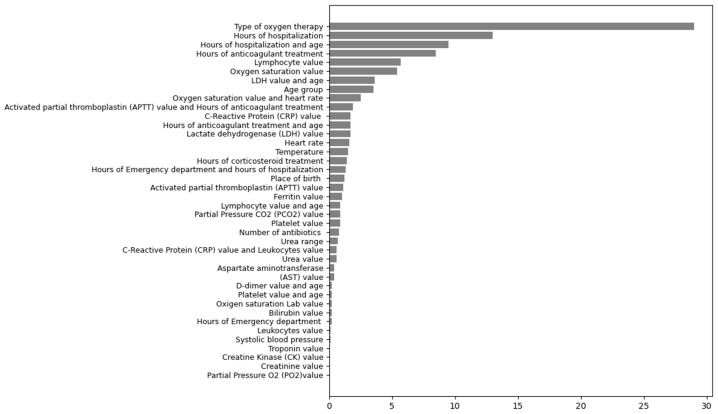
Importance of the variables (training dataset).

Only two of the variables that made up the model were collected statically (age and place of birth), the rest were collected dynamically. The classification of the type of variables is detailed in Appendix A.1. In descending order according to their gain, the most significant variables were type of oxygen therapy, hours of hospitalization, hours of hospitalization and age, hours of anticoagulant treatment, and lymphocyte value and oxygen saturation value (Figure 2).

**Figure 3:**
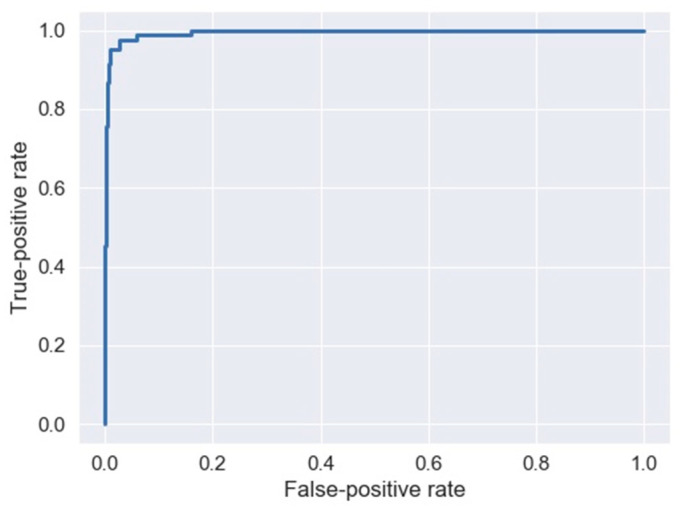
Receiver operator characteristic curve (test dataset).

The results of the model validation using the test datasets are presented in Table 3 and Figure 3. The area under the curve obtained by the LightGBM model when the test dataset was used was 1.00 (0.99–1.0), a higher value than that obtained with the training dataset 0.95 (0.93–1.00) and presenting very similar metrics in terms of specificity 0.99 (0.97–1.00) vs. 0.99 (0.98–1.00) and sensitivity 0.92 (0.86–0.98) vs. 0.91 (0.82–0.99), respectively.

**Figure 4:**
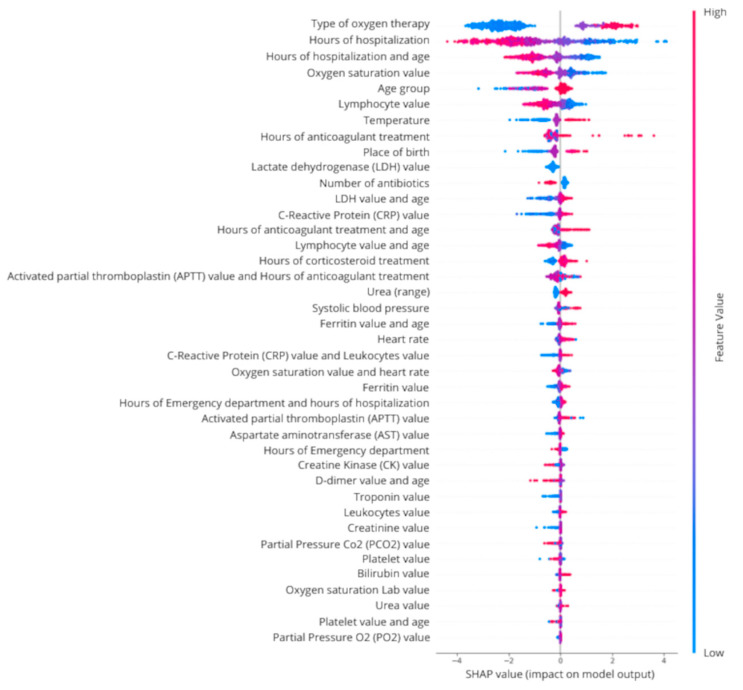
SHAP interpretation (test dataset).

Variable interpretability using SHAP was analyzed based on the test dataset. The results indicated that more intensive respiratory therapy, fewer hospitalization hours, especially in older people, low oxygen saturation, and low lymphocyte value were related to higher ICU admission risk (Figure 4).

## 4. Discussion

By identifying and prioritizing COVID-19 patients at risk of ICU admission, it is possible to provide appropriate care to those who are most vulnerable. Similarly, patients who likely do not require higher levels of care can be identified, thereby enhancing resource management efficiency during peak pressure on the health system.

Based on the above, we created and validated a model to predict ICU admission of hospitalized COVID-19 patients using machine learning techniques. The study obtained very good results.

Worthy of note, the developed model rested on variables, all of which were not collected at the same time, i.e., at admission, but on variables which were expected to be dynamic (such as laboratory results or oxygen therapy, among others), and which were collected throughout the patient’s stay. The goal was to accurately reflect the patient’s true trajectory during hospitalization. This approach prevents static databased predictions from determining the resource planning. Indeed, the disease may change in course during the provision of care: it can become more or less serious making the planning suboptimal. Moreover, the external validity of the model under study was evaluated using data from a later period.

Studies predicting ICU admission or similar outcomes, like the need for invasive mechanical ventilation, include those by Mauer et al., Cheng F.-Y. et al., Douville et al., and Park et al. Comparing the studies, our model was observed to present a superior performance to that obtained by Cheng F.-Y. et al., Douville et al., Park et al. Likewise, our model also obtained better results than those of other studies focusing on ICU admission risk in which only hospital admission time data were used [7,14,15,16,17,18]. This is unsurprising since our model was based on more accurate information about the patient’s true condition. Finally, a recent meta-analysis evaluated the joint performance of four predictive models and showed a slightly poorer overall result than that obtained in our study [8].

Regarding the variables that made up the different models which rested on the same methodology as ours, we found that all studies included variables relating to respiratory failure (e.g., respiratory rate, oxygen saturation, and type of oxygen therapy) as one of the major variables, as well as other variables linked to inflammation and/or infection, most of which were obtained from laboratory variables [3,19,20,21]. However, to the best of our knowledge, no study except the present one has included hospitalization-related variables in the final model such as “hours of hospitalization” or the derived variable “hours of hospitalization and age”, whose SHAP interpretation revealed that a lower number of hospitalization hours was related to ICU admission, especially in the elderly. Similarly, not all models included pharmacological variables in their final composition, such as the consumption of corticosteroids [21] or anticoagulants. Regarding anticoagulant treatment, the SHAP interpretation showed that a greater number of hours of treatment was related to ICU admission only in certain cases, and that in the rest, neither a greater number of hours nor fewer hours were related to this admission. In the case of corticosteroid treatment, greater use was related to ICU admission, as also mentioned in the study of Park et al.

In this work, we used a collection of dynamic variable values which reflected the patient’s actual condition and supported optimal planning according to that situation. Nevertheless, it is worth noting some study limitations. Certain variables included in previous studies, such as the National Early Warning Score (NEWS) or respiratory rate, could not be integrated in this study because they presented a high number of null values. Others, such as bilateral infiltrates, could not be incorporated because the information was not available in the EMR. Although the CRP test is the diagnostic test of reference, patients diagnosed via antigen test were included in the study. The sample size constitutes a limitation of the present study. Indeed, the number of events in the outcome (data training) was 154, which, according to the criterion of at least ten events per parameter, is lower than the minimum recommended value. However, we have calculated confidence intervals for the measures selected to appraise the model performance, which allows us to assess the accuracy of our estimates. Furthermore, the fact that the performance of the model in the dataset was even slightly higher than in the training data increases the confidence in the reliability of the results. Finally, during the study, some patients were included in clinical trials of drugs to which these researchers were blinded. Moreover, the COVID-19 vaccination campaign began during the last phase of the study and related information could not be systematically collected from the EMR.

This study has developed a machine learning-based model of ICU admission risk in COVID-19 patients, with predictors measured at admission and during the patient’s hospital stay, allowing a more realistic assessment of the patient’s condition and high predictive power. The use of SHAP techniques has facilitated the interpretability of the model, revealing the importance of predictors not previously examined in the literature, such as hours of hospitalization. The model is clinically valuable, as indicated by the fact that it has been applied routinely in the study hospitals since its validation. Its use was beneficial at the peak of the pandemic. In the current scenario, where COVID-19 coexists with other acute respiratory infections (ARI), the research team plans to re-evaluate and adjust the model for these pathologies. Thus, the model could be helpful in periods of high hospital occupancy and incidence of ARI.

## Figures and Tables

**Table 1:** Patient characteristics.

Variable	Total	%	2020 Training	%	2021 Test	%	Chi^2^, *t*-Student or Comparison of Two Population Means	*p* Value
Age (categorized)								
0–20	19	0.90	15	1.18	4	0.73		
21–40	124	5.85	90	7.08	22	4.00		
41–60	583	27.50	361	28.38	138	25.09		
61–75	832	39.24	504	39.62	228	41.45		
76–85	427	20.14	239	18.79	116	21.09		
86–150	135	6.37	63	4.95	42	7.64		
	2120	100.00	1272	100.00	550	100.00	14.34	0.0136 *
Sex								
Male	1220	57.55	741	58.26	316	57.45		
Female	900	42.45	531	41.74	234	42.55	0.0854	0.7702
	2120	100.00	1272	100.00	550	100.00		
Place of birth								
Spain	1230	58.02	855	67.22	375	68.1		
Outside Spain	592	27.92	417	32.78	175	31.81		
	2120	100.00	1272	100.00	550	100.00	0.1219	0.7269
Emergency hours								
1st Qu			5.00		2.00			
Mean (SD)			13.19 (10.85)		8.68 (7.83)		−41.065 (−43.73; −39.74)	<0.0001 *
3rd Qu			21.00		12.00			
Hours of hospitalization								
1st Qu			24.00		24.00			
Mean (SD)			207.86 (146.29)		202.00 (166.16)		0.751 (−9.42; 21.12)	0.4523
3rd Qu			261.2		258.00			
Type of oxygen therapy								
No oxygen	682	32.17	452	35.53	141	25.64		
Nasal prong	912	43.02	535	42.07	244	44.36		
Venturi oxygen mask	231	10.90	116	9.12	63	11.45		
Reservoir	193	9.10	120	9.43	64	11.64		
High flow	62	2.92	22	1.73	28	5.09		
Non-invasive mechanical ventilation	40	1.89	27	2.12	10	1.82		
	2120	100.00	1272	100.00	550	100.00	31.995	<0.0001 *
Number of antibiotics								
0	1256	59.26	704	55.36	413	75.10		
1	771	36.37	539	42.37	129	23.45		
2	76	3.57	27	2.12	8	1.45		
3	9	0.42	2	0.15	0	0.00		
4	6	0.28	0	0.00	0	0.00		
5	1	0.05	0	0.00	0	0.00		
8	1	0.05	0	0.00	0	0.00		
	2120	100.00	1272	100.00	550	100.00	63.664	<0.0001 *
Hours of anticoagulant treatment								
1st Qu			25.00		13.50			
Mean (SD)			66.75 (47.82)		63.34 (60.16)			
3rd Qu			103.00		92.00		1.2814 (−1.79; 8.58)	0.2000
Hours of corticosteroid treatment								
1st Qu			0.00		0.00			
Mean (SD)			27.12 (42.83)		51.53 (47.57)			
3rd Qu			55.00		80.00		−10.789 (−28.84; −19.97)	<0.0001 *
Systolic blood pressure								
1st Qu			110.00		115.00			
Mean (SD)			124.50 (16.47)		126.20 (16.17)			
3rd Qu			135.00		140.00		−1.9996 (−3.31; −0.03)	0.0457 *
Heart rate								
1st Qu			67.00		66.00			
Mean (SD)			77.23 (12.74)		75.44 (13.26)			
3rd Qu			86.00		82.00		2.7197 (0.50; 3.08)	0.0066 *
Temperature								
1st Qu			36.00		35.00			
Mean (SD)			36.03 (0.75)		35.80 (0.74)			
3rd Qu			39.00		36.00		5.1661 (0.13; 0.28)	<0.0001 *
Urea value								
1st Qu			29.00		36.00			
Mean (SD)			44.83 (27.47)		54.97 (32.30)			
3rd Qu			63.00		63.00		−6.8451 (−13.04; −7.23)	<0.0001 *
Bilirubin value								
1st Qu			0.40		0.30			
Mean (SD)			0.63 (0.49)		0.45 (0.35)			
3rd Qu			0.70		0.50		8.1922 (0.13; 0.22)	<0.0001 *
Creatine Kinase (CK) value								
1st Qu			46.00		63.00			
Mean (SD)			170.61 (230.82)		138.79 (145.72)		2.9863 (10.92; 52.73)	0.0029 *
3rd Qu			160.0		150.60			
Creatinine value								
1st Qu			0.69		0.71			
Mean (SD)			0.98 (0.76)		0.99 (0.86)			
3rd Qu			1.03		1.01		−0.2779 (−0.09; 0.07)	0.7917
D-dimer value								
1st Qu			425		484			
Mean (SD)			1621 (3833.99)		1920 (4688)		−1.0543 (−0.15; 0.04)	0.292
3rd Qu			1621		1920			
Ferritin value								
1st Qu			258		304			
Mean (SD)			887.8 (1041.45)		831.89 (797.67)			
3rd Qu			1197		1014		0.0464 (−0.09: 0.10)	0.963
Lactate dehydrogenase (LDH) value								
1st Qu			407		454			
Mean (SD)			565.6 (286.26)		623.32 (353.85)		−5.1556 (−0.13; −0.06)	
3rd Qu			633		687			<0.0001 *
Leukocytes value								
1st Qu			4.99		5.85			
Mean (SD)			7.31 (4.22)		8.67 (4.01)			
3rd Qu			8.71		10.51		−6.5963 (−78; −0.96)	<0.0001 *
Partial pressure CO_2_ (PCO_2_)								
1st Qu			33.10		35.20			
Mean (SD)			41.30 (8.60)		41.53 (9.33)			
3rd Qu			45.55		45.45		−0.496 (−1.14; 0.68)	0.62
Partial pressure O_2_ (PO_2_)								
1st Qu			38.38		55.95			
Mean (SD)			5976 (26.77)		63.59 (12.79)			
3rd Qu			72.20		69.05		−4.1106 (−8.47; −2.99)	<0.0001 *
Platelet value								
1st Qu			182		176			
Mean (SD)			258 (108.97)		254 (110.66)			
3rd Qu			312		323		0.9963 (−0.02; 0.07)	0.3194
Troponin value								
1st Qu			0.017		0.008			
Mean (SD)			0.03 (0.07)		0.05 (0.073)			
3rd Qu			0.03		0.046		−4.0135 (−0.34; −0.12)	<0.0001 *
Aspartate aminotransferase (AST) value								
1st Qu			27		27			
Mean (SD)			46 (33.92)		44 (28.71)		1.1084 (−1.32; 4.75)	0.2679
3rd Qu			54		50			
Activated partial thromboplastin (aPTT) value								
1st Qu			24.40		22.30			
Mean (SD)			29.94 (7.50)		26.74 (9.69)			
3rd Qu			34.40		30.94		8.2431 (2.14; 3.47)	<0.0001 *

Note: * Statistical difference with a *p*-value less than 0.05.

**Table 2:** Performance of the different predictive classification models.

	PPV (95% CI)	NPV (95% CI)	Accuracy (95% CI)	AUC (95% CI)	Specificity (95% CI)	Sensitivity (95% CI)	F1-S	Youden Index
LightGBM	0.93 (0.85–1.0)	0.99 (0.98–1.0)	0.98 (0.97–1.0)	0.95 (0.93–0.97)	0.99 (0.98–1.0)	0.91 (0.82–0.99)	0.94	0.91
XGBoost	0.93 (0.85–1.0)	0.99 (0.98–1.0)	0.98 (0.97–1.0)	0.94 (0.91–0.97)	0.99 (0.98–1.0)	0.91 (0.82–0.99)	0.91	0.91
Logistic regression	0.85 (0.75–0.96)	0.97 (0.96–0.99)	0.98 (0.97–1.0)	0.92 (0.89–0.95)	0.98 (0.97–1.0)	0.80 (0.68–0.91)	0.79	0.81
Random Forest	0.95 (0.88–1.0)	0.98 (0.96–1.0)	0.96 (0.94–0.98)	0.90 (0.87–0.93)	0.99 (0.98–1.0)	0.84 (0.73–0.95)	0.88	0.93

Note: PPV (positive predictive value), NPV (negative predictive value), accuracy, AUC (area under the curve), F1-S (F1-score).

**Table 3:** Results of the models using training dataset and test dataset.

	PPV (95% CI)	NPV (95% CI)	Accuracy (95% CI)	AUC (95% CI)	Specificity (95% CI)	Sensitivity (95% CI)	F1-S	Youden Index
LightGBM (Training)	0.93 (0.85–1.00)	0.99 (0.97–1.00)	0.98 (0.97–1.00)	0.95 (0.93–1.00)	0.99 (0.98–1.00)	0.91 (0.82–0.99)	0.94	0.91
LightGBM (Test)	0.95 (0.90–1.00)	0.99 (0.98–1.00)	0.98 (0.97–0.99)	1.00 (0.99–1.00)	0.99 (0.97–1.00)	0.92 (0.86–0.98)	0.93	0.93

Note: PPV (Positive Predictive Value), NPV (Negative Predictive Value), AUC (Area Under the Curve), F1-S (F1 Score).

## Data Availability

Data is unavailable due to privacy restrictions.

## Abbreviations

The following abbreviations are used in this manuscript:

ICU	Intensive Care Unit
EMR	Electronic Medical Record
CRP	C-Reactive Protein
RFE	Recursive Feature Elimination
IQR	Interquartile range
RFECV	Recursive Feature Elimination Cross Validation
ADASYN	Adaptive synthetic sampling approach for imbalanced learning
AUC	Area Under the Curve
SHAP	Shapley Additive Explanations
AST	Aspartate aminotransferase
NEWS	National Early Warning Score
ARI	Acute Respiratory Infections
LDH	Lactate dehydrogenase
PCO_2_	Partial pressure CO_2_
PO_2_	Partial pressure O_2_
aPTT	Activated partial thromboplastin
PPV	Positive Predictive Value
NPV	Negative Predictive Value
F1-S	F1-Score

## Appendix A

### Appendix A.1

**Table A1:** Model variables.

Variable	No. Values	Type of Value	Type of Variable	Data Collection Timing
Number of antibiotics	8	Accumulated	Dynamic	Value prior to ICU admission or expected ICU admission (median)
Hours of anticoagulant treatment	All	Accumulated	Dynamic	Value prior to ICU admission or expected ICU admission (median)
Aspartate aminotransferase (AST) value	All	Individual	Dynamic	Last recorded value prior to ICU admission or expected ICU admission (median)
Bilirubin value	All	Individual	Dynamic	Last recorded value prior to ICU admission or expected ICU admission (median)
Creatine Kinase (CK) value	All	Individual	Dynamic	Last recorded value prior to ICU admission or expected ICU admission (median)
Hours of corticosteroid treatment	All	Accumulated	Dynamic	Value prior to ICU admission or expected ICU admission (median)
Creatinine value	All	Individual	Dynamic	Last recorded value prior to ICU admission or expected ICU admission (median)
D-dimer value	All	Individual	Dynamic	Last recorded value prior to ICU admission or expected ICU admission (median)
Ferritin value	All	Individual	Dynamic	Last recorded value prior to ICU admission or expected ICU admission (median)
Heart rate	All	Individual	Dynamic	Last recorded value in the 24 h prior to ICU admission or expected ICU admission (median)
Age group	6	Individual	Static	On admission
Hours of hospitalization	All	Accumulated	Dynamic	Value from the emergency department admission to the ICU admission or to the discharge day
Hours of Emergency department	All	Accumulated	Dynamic	Value from the emergency department stay
Lactate dehydrogenase (LDH) value	All	Individual	Dynamic	Last recorded value prior to ICU admission or expected ICU admission (median)
Leukocytes value	All	Individual	Dynamic	Last recorded value prior to ICU admission or expected ICU admission (median)
Lymphocyte value	All	Individual	Dynamic	Last recorded value prior to ICU admission or expected ICU admission (median)
Place of birth	2	Individual	Static	On admission
Type of oxygen therapy	6	Individual	Dynamic	Last recorded value in the 24 h prior to ICU admission or expected ICU admission (median)
Partial Pressure CO_2_ (PCO_2_) value	All	Individual	Dynamic	Last recorded value prior to ICU admission or expected ICU admission (median)
Platelet value	All	Individual	Dynamic	Last recorded value prior to ICU admission or expected ICU admission (median)
Partial Pressure O_2_ (PO_2_) value	All	Individual	Dynamic	Last recorded value prior to ICU admission or expected ICU admission (median)
C-Reactive Protein (CRP) value	All	Individual	Dynamic	Last recorded value prior to ICU admission or expected ICU admission (median)
Urea range	2	Individual	Dynamic	Last recorded value prior to ICU admission or expected ICU admission (median)
Oxygen saturation value	All	Individual	Dynamic	Last recorded value in the 24 h prior to ICU admission or expected ICU admission (median)
Oxygen saturation Lab value	All	Individual	Dynamic	Last recorded value prior to ICU admission or expected ICU admission (median)
Temperature	All	Individual	Dynamic	Last recorded value in the 24 h prior to ICU admission or expected ICU admission (median)
Systolic blood pressure	All	Individual	Dynamic	Last recorded value in the 24 h prior to ICU admission or expected ICU admission (median)
Troponin value	All	Individual	Dynamic	Last recorded value prior to ICU admission or expected ICU admission (median)
Activated partial thromboplastin (aPTT) value	All	Individual	Dynamic	Last recorded value prior to ICU admission or expected ICU admission (median)
Urea value	All	Individual	Dynamic	Last recorded value prior to ICU admission or expected ICU admission (median)

### Appendix A.2

**Table A2:** Hyperparameters setting for the LightGBM model.

Hyperparameter	Value
boosting_type	gbdt
class_weight	balanced
colsample_bytree	0.4750764935387468
importance_type	split
learning_rate	0.28312968303160313
max_depth	−1
min_child_samples	3
min_child_weight	0.001
min_split_gain	0.0
n_estimators	76
n_jobs	4
num_leaves	326
objective	binary
random_state	50
reg_alpha	0.5985119228590867
reg_lambda	0.5845451820124326
silent	True
subsample	1.0
subsample_for_bin	300000
subsample_freq	0
bagging_fraction	0.6087654298259604
